# Food insecurity and self-reported cholera in Haitian households: An analysis of the 2012 Demographic and Health Survey

**DOI:** 10.1371/journal.pntd.0007134

**Published:** 2019-01-30

**Authors:** Aaron Richterman, Molly F. Franke, Georgery Constant, Gregory Jerome, Ralph Ternier, Louise C. Ivers

**Affiliations:** 1 Department of Medicine, Brigham & Women’s Hospital, Boston, Massachusetts, United States of America; 2 Department of Global Health and Social Medicine, Harvard Medical School, Boston, Massachusetts, United States of America; 3 Zanmi Lasante / Partners In Health, Cange, Haiti; 4 Massachusetts General Hospital, Center for Global Health, Boston, Massachusetts, United States of America; Johns Hopkins University Bloomberg School of Public Health, UNITED STATES

## Abstract

**Background:**

Both cholera and food insecurity tend to occur in impoverished communities where poor access to food, inadequate sanitation, and an unsafe water supply often coexist. The relationship between the two, however, has not been well-characterized.

**Methods:**

We performed a secondary analysis of household-level data from the 2012 Demographic and Health Survey in Haiti, a nationally and sub-nationally representative cross-sectional household survey conducted every five years. We used multivariable logistic regression to evaluate the relationship between household food security (as measured by the Household Hunger Scale) and (1) reported history of cholera since 2010 by any person in the household and (2) reported death by any person in the household from cholera (among households reporting at least one case). We performed a complete case analysis because there were <1% missing data for all variables.

**Results:**

There were 13,181 households in the survey, 2,104 of which reported at least one household member with history of cholera. After adjustment for potential confounders, both moderate hunger in the household [Adjusted Odds Ratio (AOR) 1.51, 95% Confidence Interval (CI) 1.30–1.76; p <.0001] and severe hunger in the household (AOR 1.73, 95% CI 1.45–2.08; p <.0001) were significantly associated with reported history of cholera in the household. Severe hunger in the household (AOR 1.85, 95% CI 1.05–3.26; p = 0.03), but not moderate hunger in the household, was independently associated with reported death from cholera in households with at least one case of cholera.

**Conclusions:**

In this study we identified an independent relationship between household food insecurity and both reported history of cholera and death from cholera in a general population. The directionality of this relationship is uncertain and should be further explored in future prospective research.

## Author summary

In this study, we identified an independent relationship between household food insecurity, defined as a persistent lack of access to food in adequate quantity or quality and measured using the Household Hunger Scale, and reported history of cholera and death from cholera in a general population. We performed a secondary analysis of household-level data from the 2012 Demographic and Health Survey (DHS) in Haiti, a nationally and sub-nationally representative cross-sectional household survey conducted every five years. The 2012 survey was conducted during the height of the cholera epidemic, with 453,536 suspected cases and 3,835 deaths in Haiti from 2011–2012. We used multivariable logistic regression to control for measured confounders. The underlying mechanisms and directionality of the association between food insecurity and reported history of cholera are uncertain and should be explored in future prospective research. A better understanding of the relationship between food insecurity and cholera could inform both future cholera outbreak prediction and response, particularly in settings where poor food access and cholera risk factors are known to co-exist.

## Introduction

Food insecurity is defined as a persistent lack of access to food in adequate quantity or quality [1]. Food insecurity is linked to, and often results from, poverty [2], which is associated with poor health outcomes including mortality [3–6]. Food insecurity itself is also associated with an increased risk of death, even after controlling for neighborhood or household wealth and other social determinants of health [7]. Food insecurity has been specifically linked to poor health outcomes for patients with a variety of conditions including Human Immunodeficiency Virus (HIV) infection and cardiovascular disease [1]. While the effect of food insecurity on health is often thought of as synonymous with malnutrition, which has profound short and long-term health consequences [8], it has also been implicated in a number of other pathways which impact health. These include behavioral pathways, like clinical follow up, medication adherence, and substance use; and mental health pathways, like depression and anxiety [1, 9–14]. Food insecurity is also associated with chronic inflammation [15–17], which in turn is related to worse health outcomes in a number of conditions including infectious diseases like HIV and tuberculosis (TB) [18, 19].

The relationship between health and food insecurity has in many cases been found to be bi-directional, with poor health outcomes increasing subsequent risk of food insecurity [1, 9]. This creates the potential for a vicious cycle and may result from the impoverishing effects of healthcare utilization and illness.

Cholera remains a major cause of morbidity and mortality worldwide and is now endemic in Haiti since being inadvertently introduced in 2010 [20, 21]. A wide variety of potential risk factors for cholera, including poverty, have been identified and inform institutional guidance during the response to a cholera outbreak [22, 23]. The relationship between food insecurity and cholera, however, has not been well studied despite the fact that both cholera outbreaks and food insecurity tend to occur in impoverished communities where poor access to food, inadequate sanitation, and an unsafe water supply often co-exist [1, 20]. The most recent example of this confluence is in Yemen, where 15 million people are estimated to lack access to safe water and sanitation, at least 17 million people are food insecure, and more than 700,000 cases of cholera along with 2,000 deaths from cholera have been reported since 2016 [24]. As in other health-related settings, it is plausible that food insecurity may increase risk of cholera, or the severity of cholera, through multiple pathways–including malnutrition, by impairing immune or gut barrier function [25, 26]; behavioral pressures, by increasing the likelihood of drinking unsafe water or eating unsafe food; by impacting mental health; or through other mechanisms. An episode of cholera has also been found to cause significant financial strain on a household [27–29], and may increase the downstream risk of food insecurity.

In a recent analysis of HIV-affected households in rural Haiti we found that household food insecurity was independently associated with reported history of cholera [30]. However, food insecurity and HIV-related outcomes are closely linked [9, 13, 14, 17, 31], and thus it is unknown whether this relationship exists among HIV-unaffected households. In this study we sought to answer this question by exploring the relationship between risk of cholera and food insecurity in a general population, using data from the 2012 Demographic and Health Survey (DHS) in Haiti [32].

## Methods

### Data

This study is a secondary analysis of household-level data from the 2012 DHS in Haiti, a nationally and sub-nationally representative cross-sectional household survey conducted every five years by the Ministry of Public Health and Population with support from Independent Consulting Firm International [32]. The DHS survey sampling methods have been previously described [33]. The 2012 DHS in Haiti used a two-stage cluster sampling design to produce indicator estimates for the ten administrative departments of Haiti, the capital region, and for internally-displaced person (IDP) camps resulting from the earthquake in 2010 [32]. In the first stage, 445 clusters (144 urban, 256 rural, 45 IDP camps) were systematically selected with probability proportional to population size. This was done by selecting clusters at a fixed interval (with likelihood of selection of each cluster proportional to its population size) from a randomly determined starting point on a list of all the Enumeration Areas established by the Fourth General Census of Housing and Population in 2003. In the second stage, a systematic sample of households was drawn from each of these clusters by selecting them at a fixed interval from a randomly determined starting point on a list of all households within a given cluster for a total of 13,388 households, of which 13,181 were successfully surveyed. The primary survey respondents were female heads of household.

Because of the ongoing cholera epidemic in Haiti at the time, the DHS assessed history of cholera by asking the primary survey respondent how many household members had cholera since October 2010, and whether those with a reported history died as a result of the illness (Supporting Information Table 1). Food security was assessed using the Household Hunger Scale, a validated subset of three items from the Household Food Insecurity Access Scale (HFIAS) which has been shown to be culturally invariant (Table 1) [34]. The Household Hunger Scale classifies food security into one of three categories: little to no hunger in the household (score of 0–1), moderate hunger in the household (score of 2–3), or severe hunger in the household (score of 4–6).

**Table 1:** The household hunger Scale assesses the presence and severity of food insecurity using three questions from the Household Food Insecurity Access Scale (HFIAS) which have been shown to be culturally invariant. Food security is classified into one of three categories: little to no hunger in the household (score of 0–1), moderate hunger in the household (score of 2–3), or severe hunger in the household (score of 4–6) [34].

Question	Score
In the past four weeks, how often was there no food of any kind to eat in the household?	Never (0); Sometimes (1); Often (2)
In the past four weeks, how often did one or more household members go to sleep hungry?	Never (0); Sometimes (1); Often (2)
In the past four weeks, how often did one or more household members spend an entire day and night without eating?	Never (0); Sometimes (1); Often (2)

Procedures and questionnaires for DHS surveys have been reviewed and approved by the Independent Consulting Firm Institutional Review Board (IRB), and all analyzed data were anonymized.

### Analysis

We used multivariable logistic regression to evaluate the association between food security in the household and (1) reported history of cholera by any person in the household and (2) reported death from cholera by any person in the household (among households with at least one reported case of cholera). Based on published literature, we identified twelve potential confounders measured by the DHS: rural/urban setting; number of household members; number of children under five years old; possession of land usable for agriculture; possession of livestock, herds, or farm animals; primary household roof material; primary household floor material; improved source of drinking water (piped household water, protected wells or springs, collected rainwater); time required to reach a water source; access to a latrine; number of rooms for sleeping; and wealth index. The wealth index is a composite measure of a household’s cumulative living standard calculated by the DHS using ownership of certain assets, materials used for housing construction, and types of water access and sanitation facilities.

We conducted a complete case analysis because there were 2 (0.02%) households missing the Household Hunger Scale, none missing cholera outcomes, and <1% missing all other covariates of interest. We first calculated unadjusted odds ratios (OR) with 95% confidence intervals (CI) between the Household Hunger Scale and reported history of cholera and reported death from cholera using bivariate logistic regression models. We then used bivariate logistic regression models to identify which of the previously listed covariates were correlated with both the relevant outcome and the Household Hunger Scale with p<0.2 and included these variables in multivariable logistic regression analyses. We did not use backwards or stepwise elimination. If both the wealth index and a component of the wealth index met criteria for inclusion in the model, only the wealth index was included. The Household Hunger Scale was modeled as moderate hunger in the household compared to no hunger in the household and severe hunger in the household compared to no hunger in the household. We calculated the variance inflation factor to assess for multicollinearity among model covariates. An inflation factor greater than 2.50 was considered indicative of multicollinearity.

We performed statistical analysis using SAS version 9.4 (SAS Institute, Cary, North Carolina), using survey commands to apply sampling probability weights and account for clustering and stratification in the sample design.

## Results

The DHS surveyed 13,181 households which contained a median of 4.4 (IQR 2.8–6.2) household members. Of these households, 2,104 reported at least one household member with a history of cholera since 2010 (Table 2). There was a mean of 1.35 (SD 1.02) people with reported history of cholera per household among households reporting at least one, and 151 (1.1%) households reporting history of cholera also reported at least one member who had died as a result. Households reporting at least one person with history of cholera were significantly more likely to be located in a rural zone, own land usable for agriculture, and own animals. These households were significantly less likely to have access to an improved water source or latrine. They also had more household members, were more likely to have an earth floor, and had less wealth.

**Table 2:** Characteristics of households participating in the 2012 DHS in Haiti grouped by whether they reported at least one member with history of cholera since 2010 (N = 13,181*).

		Reported History of Cholera in Household	No Reported History of Cholera in Household	
		Weighted N = 2104^θ^	Weighted N = 11077^θ^	p-value^Ψ^
**Rural**		1503 (71.4)	6264 (56.6)	<.0001
**Number household members, median (IQR)**		4.4 (2.7–6.2)	3.5 (2.0–5.2)	<.0001
**Wealth quintile**	Poorest	579 (27.5)	1763 (15.9)	<.0001
	Poorer	567 (27.0)	2114 (19.1)	
	Middle	468 (22.2)	2406 (21.7)	
	Richer	361 (17.2)	2422 (21.9)	
	Richest	128 (6.1)	2371 (21.4)	
**Owns usable land for agriculture (Weighted N = 13180)**		1567 (74.5)	6687 (60.4)	<.0001
**Owns livestock, herds, or farm animals**		1294 (61.5)	5407 (48.8)	<.0001
**Improved drinking water source**^§^ **(Weighted N = 13102)**		1088 (51.8)	7925 (72.0)	<.0001
**Time required to get drinking water, median (IQR)**		19.8 (9.5–55.7)	18.3 (9.1–42.6)	0.2943
**Access to latrine (Weighted N = 13146)**		1271 (60.6)	8385 (75.9)	<.0001
**Number of rooms for sleeping, median (IQR)**		1.15 (1.00–1.79)	1.02 (0.51–1.74)	0.01
**Primary floor material**	Earth	1154 (54.8)	3895 (35.2)	<.0001
	Cement/Concrete/Carpet	901 (42.8)	6406 (57.8)	
	Ceramic/Wood	49 (2.3)	775 (7.0)	
**Primary roof material**	Cement	187 (8.9)	2266 (20.5)	<.0001
	Metal	1578 (75.0)	7471 (67.4)	
	Other	339 (16.1)	1339 (12.1)	
**Household Hunger Scale (Weighted N = 13179)**	Little to no hunger	570 (27.1)	4728 (42.7)	<.0001
	Moderate hunger in household	959 (45.6)	4182 (37.8)	
	Severe hunger in household	576 (27.3)	2165 (19.5)	
**Number of household members with reported history of cholera, mean (SD)**		1.35 (1.02)	n/a	
**One or more reported death from cholera in household (Weighted N = 13180)**		151 (1.1)	n/a	

IQR, Interquartile range

Data are presented as N (%) unless otherwise specified.

* Unless otherwise noted

^θ^ Weighted by sampling probability weights

^Ψ^ P-values are from Fisher’s exact tests for proportional variables and Student t tests for continuous variables

^§^ Tap, covered, protected spring, rain water, or purchased

Table 3 shows the unadjusted and adjusted relationship between food security and (1) reported history of cholera in the household and (2) reported history of death from cholera in the household (among households with at least one reported case of cholera). In unadjusted analysis, relative to households with little to no hunger, both moderate hunger in the household (OR 1.90, 95% CI 1.66–2.19; p <.0001) and severe hunger in the household (OR 2.21, 95% CI 1.85–2.63; p <.0001) were significantly associated with reported history of cholera in the household. Among households reporting at least one cholera case, severe hunger in the household (OR 1.87, 95% CI 1.05–3.34; p = 0.03) was associated with at least one death from cholera in the household relative to households with little to no hunger.

**Table 3:** Unadjusted and adjusted relationships between food security and (1) reported history of cholera by any member of the household since 2010 (Weighted N = 13,061), (2) reported history of death from cholera by any member of the household since 2010 among households reporting at least one cholera case (Weighted N = 2,104).

***(1) Any reported history of cholera in the household***							
		*Unadjusted*			*Adjusted*^a^		
		Odds Ratio	95% CI	p-value	Odds Ratio	95% CI	p-value
Household Hunger Scale	Little to no hunger in household	ref			ref		
	Moderate hunger in household	1.90	1.66–2.19	<.0001	1.51	1.30–1.76	<.0001
	Severe hunger in household	2.21	1.85–2.63	<.0001	1.73	1.45–2.08	<.0001
***(2) Any reported history of death from cholera in the household (among households reporting at least one cholera case)***							
		*Unadjusted*			*Adjusted*^b^		
		Odds Ratio	95% CI	p-value	Odds Ratio	95% CI	p-value
Household Hunger Scale	Little to no hunger in household	ref			ref		
	Moderate hunger in household	1.03	0.61–1.75	0.91	1.02	0.60–1.71	0.95
	Severe hunger in household	1.87	1.05–3.34	0.03	1.85	1.05–3.26	0.03

CI, confidence interval; ref, reference

^a^ The multivariable model includes the Household Hunger Scale, urban or rural setting, number of children age five or less in the household, wealth index, and number of rooms in the house for sleeping.

^b^ The multivariable model includes the Household Hunger Scale and wealth index.

The multivariable model for the relationship between food security and reported history of cholera in the household included the Household Hunger Scale, urban or rural setting, number of children age five or less in the household, wealth index, and number of rooms for sleeping. Compared to households with little to no hunger, both moderate hunger in the household [Adjusted Odds Ratio (AOR) 1.51, 95% Confidence Interval (CI) 1.30–1.76; p <.0001] and severe hunger in the household (AOR 1.73, 95% CI 1.45–2.08; p <.0001) were significantly associated with reported history of cholera in the household.

The multivariable model for the relationship between food security and reported history of death from cholera in the household included the wealth index. Severe hunger in the household (AOR 1.85, 95% CI 1.05–3.26; p = 0.03), but not moderate hunger in the household (AOR 1.02, 95% CI 0.60–1.71; p = 0.95), was significantly associated with reported death from cholera compared to little or no hunger in the household. There was no evidence of multicollinearity in either multivariable model.

## Discussion

In this analysis of 13,181 households surveyed in the 2012 DHS in Haiti, we found moderate and severe household food insecurity to be independently associated with a reported history of cholera, and severe household food insecurity to be independently associated with a reported history of death from cholera among households with at least one reported case of cholera. The DHS was conducted during the peak of the cholera epidemic, with 453,536 suspected cases and 3,835 deaths in Haiti from 2011–2012 [35].

We previously found that food insecurity was associated with a reported history of cholera in a multivariable analysis of 352 HIV-affected households in Haiti [30]. However, there is a well-documented relationship between HIV-related morbidity and mortality and food insecurity [9, 14, 31], and the generalizability of these findings to HIV-unaffected populations was uncertain. Another case-control study in Haiti found that a diverse diet, which is one element of food security [36], was independently associated with a decreased risk of cholera [37]. In this study we have now identified an independent relationship between household food insecurity and both reported history of cholera and death from cholera in a general population in Haiti.

Because of the cross-sectional nature of the DHS it was not possible to determine the temporal relationship between food insecurity and reported history of cholera and thus the directionality of this association is unknown. In other health settings food insecurity has been found to exist both upstream and downstream of disease morbidity and mortality, in a vicious cycle [1, 9], and a similar framework may be true in the setting of cholera.

Food insecurity may increase risk of cholera in several ways. Malnutrition as a result of food insecurity could directly increase the risk of cholera infection by impairing immune and gut barrier function [25, 26]. Food insecurity might also increase risk of cholera indirectly, by impacting behavior. Just as food insecurity increases high-risk practices in the setting of HIV [12], it is plausible that people living in food insecure households are more likely to engage in behaviors that increase their risk of cholera, including drinking from unsafe water sources or consuming unsafe food.

On the other hand, an episode of cholera in a household may also increase subsequent risk of food insecurity, in part as a result of the direct and indirect costs of illness and healthcare utilization. Despite the typically short duration of illness, cholera has been found to have substantial household costs in both the epidemic and endemic setting [27–29].

In addition to the link between risk of cholera and food insecurity, our findings also suggest an independent relationship between food insecurity and cholera-related mortality. Again, the temporality and direction of this relationship is uncertain and may plausibly be bidirectional. People with cholera in severely food insecure households may have increased risk of death compared to those in food secure households because of more severe illness in the setting of malnutrition, decreased ability to seek or access care, worse mental health status at baseline that may impact response to acute illness, or other unknown mechanisms. On the other hand, households in which a member dies as a result of cholera are likely to incur magnified costs because of funeral expenses and a permanent loss in contribution to household income which may decrease household economic resilience and increase risk of subsequent food insecurity.

The strengths of this study include a rigorous sampling methodology, a large and representative sample, the availability of multiple potential cofounding covariates, little missingness of data, and a cross-culturally validated measure of food insecurity.

This study also has some limitations. As mentioned, we were unable to determine the directionality of the relationship between reported history of cholera and household food insecurity. Poverty and food insecurity are closely related, and there may be residual confounding by the impact of poverty on cholera risk which is not accounted for by the DHS wealth index. However, we believe this is likely to be minimal as the wealth index is a well-validated and reasonably comprehensive measure of socioeconomic status, Additionally, there may be other confounders of the relationship between food insecurity and cholera risk which were not measured in the DHS and thus not adjusted for in our analysis. Potential unmeasured confounders should be addressed in future research and include diet composition, hygiene practices, and household water treatment.

The DHS assessed food insecurity and history of cholera through self-report which is subject to recall bias. If some survey respondents over-reported both food insecurity and cholera, this would have inflated the estimated odds ratio relative to the true odds ratio. However, the survey was conducted shortly after the cholera epidemic peaked in Haiti, and less than two years after it began. We believe that significant episodes of diarrheal illness are likely to be remembered in a household within this time frame. Microbiologic confirmation of cholera was not available and thus some cases may have been a result of other causes of acute watery diarrhea. In any case, cholera was generally diagnosed using a standard clinical definition in Haiti at the time, in keeping with the World Health Organization case-definition of cholera during an active outbreak [38].

While this study suggests that food insecurity and cholera risk are independently related at the household level, we also note the confluence of regional food insecurity and massive cholera epidemics in Haiti and Yemen [24, 35, 39]. Future research should address whether there is also an association between regional food insecurity and risk of cholera epidemics.

In conclusion, we found a significant relationship between household food insecurity and both reported history of cholera and reported death from cholera in a large representative sample of Haitian households after adjusting for potential confounders. The underlying mechanisms and directionality of this association are uncertain and should be explored in future prospective research. A better understanding of the relationship between food insecurity and cholera could inform both future cholera outbreak prediction and response, particularly in settings where poor food access and cholera risk factors are known to co-exist [24].

## Supporting information

S1 Table Items from the 2012 Demographic and Health Survey (DHS) in Haiti which addressed history of cholera and death from cholera within the household [32]. (DOCX)

S1 Checklist STROBE Checklist. (PDF)
