# Supplementary material for: Food insecurity and self-reported cholera in Haitian households: An analysis of the 2012 Demographic and Health Survey
Source: PLoS Negl Trop Dis. 2019 Jan 30;13(1):e0007134. doi: 10.1371/journal.pntd.0007134 (PMC6370226; doi:10.1371/journal.pntd.0007134)
Supplement: S1 Table — (DOCX) [file pntd.0007134.s001.docx]

Supporting Information Table 1. Items from the 2012 Demographic and Health Survey (DHS) in Haiti which addressed history of cholera and death from cholera within the household [32].

| From October 2010 until now, has one or more person in your household gotten cholera, but not died? |
| --- |
| If yes, from October 2010 until now, how many people have gotten cholera, but not died? |
| From October 2010 until now, has one or more person in your household gotten cholera, and died because of cholera? |
| If yes, from October 2010 until now, how many people have gotten cholera, and died because of cholera? |
